# A Cross-Sectional Survey of Knowledge, Attitude, and Practices of University Students in Pakistan Regarding COVID-19

**DOI:** 10.3389/fpubh.2021.697686

**Published:** 2021-11-18

**Authors:** Sohail Raza, Nadia Mukhtar, Muhammad Nawaz, Muhammad Asad Ali, Muhammad Abu Bakr Shabbir, Muhammad Adnan Ashraf, Zeeshan Ali, Muhammad Rizwan Saleem, Rabia Latif, Tahir Yaqub

**Affiliations:** ^1^Institute of Microbiology, University of Veterinary and Animal Sciences, Lahore, Pakistan; ^2^Information Technology Center, University of Veterinary and Animal Sciences, Lahore, Pakistan; ^3^Department of Anatomy, University of Health Sciences Lahore, Lahore, Pakistan

**Keywords:** COVID-19 pandemic, KAP survey, health, education, Pakistan

## Abstract

The COVID-19 pandemic is striking the world with serious public health and socioeconomic complications. The pandemic has influenced all forms of daily life, including educational institutions. Therefore, this cross-sectional survey was conducted to understand knowledge, attitudes, and practices related to COVID-19 among the students of the University of Veterinary and Animal Sciences, Lahore. The data was collected using an online self-directed questionnaire. The survey form includes six items about sociodemographic characteristics, 14 knowledge-based questions, seven questions on attitude, and eight questions on practices. The sample number was calculated using the Raosoft sample size calculator. A total number of 3,854 students, including 1,823 men and 2,031 women, were engaged in this survey, having student representation from all the provinces in the country. The data were analyzed using a chi-square test. A total of 97% of the students knew that the etiological agent of COVID-19 is a virus and that it is a disease of the respiratory system (94%). Many students kept visiting their relatives during the lockdown (45%), and their relatives kept visiting them at home (59%). The responses from the students varied a lot on specific questions about the transmission of the virus. Women tended to have less information regarding precautionary travel measures (*p* < 0.01), but supplemental knowledge of prevention of disease transmission from positive patients (*p* < 0.01). Conclusively, the majority of the university students surveyed had imperative knowledge, a good attitude, and active practice in response to the COVID-19 outbreak. Moreover, the KAP scores have varied by demography, gender, and the number of family members. Therefore, continuous awareness of preventative behaviors should be disseminated regularly in emergencies.

## Introduction

In December 2019, an outbreak of respiratory disease erupted in Wuhan market, P.R. China. The initial few cases had the history of working in the fish market in Wuhan, where wet animals including bats were being sold (1). The outbreak is believed to have started from the transmission of the virus from animals to humans. The disease was very contagious and human-to-human spread was very swift. The virus was preliminarily named as a novel coronavirus (n-CoV) which was renamed as severe acute respiratory syndrome coronavirus 2 (SARS-CoV-2) (2). The disease spread to 18 countries within 2 months which prompted the World Health Organization (WHO) to declare it as Public Health of International Concern on January 30, 2020 (3). The number of cases persistently increased, which resulted in the WHO declaring it as a pandemic on March 11, 2020. As of April 17, 2021, the total number of people who have been infected with COVID-19 is 140,598,841 while 3,014,240 people have died of the disease. The cases in Pakistan increased rapidly as well with the first case reported on February 26, 2020 (4), while 750,158 cases have been reported and 16,094 people have lost their lives to COVID-19 as of April 17, 2021.

The infectious period of COVID-19 has not been completely elucidated at the time of this study, and the incubation period of SARS-CoV-2 varies from 5 to 14 days (5). The virus affects the lower respiratory tract leading to pneumonia although it can also affect the gastrointestinal tract, liver, kidney, and multiple organs (6). Scientists from multiple countries have collaborated under the Coalition for Epidemic Preparedness Innovations (CEPI) on the development of a vaccine. Many scientists have worked on the repurposing of already available drugs. Approval and availability of vaccines and drugs require time to ensure their safety before mass production and widespread use (7). Some antiviral drugs including hydroxychloroquine, favipiravir, lopinavir, and remdesivir are being tested in Phase III and Phase IV trials. Currently, the most effective way to prevent the spread of disease is to follow the Standard Operating Procedures and guidelines shared by the WHO and the Center for Disease Control (CDC).

At time of writing, Pakistan is tackling the fourth wave of COVID-19. The positivity rate of COVID-19 varies from 4 to 11 percent. The management strategies include awareness campaigns regarding the transmission and prevention of the disease. Before vaccine availability, the smart lockdown strategy of Pakistan has let the economy keep moving and kept the disease under control. Cities showing high positivity rates are brought under lockdown, and public gatherings are banned. The intermittent lockdown strategy is one of the success stories of Pakistan. After vaccine availability, the mass vaccination of people has played its role in controlling of the disease. A total of 25,493,964 people in Pakistan have at present been completely vaccinated. Nevertheless, much of the population does not believe in the existence of coronavirus, therefore, there is a continuous need to educate people both conventionally and non-conventionally.

In Pakistan, the National Institute of Health has also shared the guidelines and arranged training sessions to strengthen preventive measures, which emphasized frequent hand washing, mandatory face mask use, and maintaining social distancing. Government and non-government organizations and print, electronic, and social media have done massive awareness campaigns regarding prevention measures (8). Nevertheless, the cultural norms of Pakistan having a cordial community make it difficult to maintain social distancing over a longer period of time. As with the disease spreading fast, scientific data evolved rapidly on a daily basis. On another aspect, there is mixed information available the disease, further much of the incorrect information available through social media creates an “infodemic”, which develops unnecessary fear among the community (9).

It is evident from previous pandemics that lack of knowledge and intention to practice SOPs mitigates the efforts to control the spread of disease (10). Several previous studies based on the KAP survey provide insight into COVID-19 information and practices of the students of universities, colleges, and general people in society. This helps policymakers to make efficient policy considering the results of the survey in order to control the spread of the disease (11–13). Amidst the infodemic, Universities are opening, although, the battle is still going on against COVID-19. Multiple studies have been done to know the response of the community to the COVID-19 disease, but few studies were planned for the students of universities. University students can reflect the view of the educated youth of diverse communities. A delicate balance between the Knowledge, Attitude, and Practices (KAP) of a community is required; therefore, there was the need for a survey that could reflect students' understanding of COVID-19 during December 2020. The perceived efficacy of KAP will help to underpin the thorough guidelines and SOPs for universities. This may help the government to devise effective SOPs and awareness campaigns.

## Materials and Methods

### Study Designs and Participants

A cross-sectional study was conducted in December 2020 among the students of the University of Veterinary and Animal Sciences (UVAS), Lahore with approval. The collection of the data was according to the regulations of the Institutional Ethical committee based on the Helsinki declaration. Anonymity was ensured while the collection of information. The sampling technique for the study was non-probability convenience sampling. Data were obtained from the students of UVAS, Lahore. The campus is located in the heart of Lahore city, which is the capital of the Punjab province, Pakistan. The students of university have inclusiveness from all the provinces along with representation from foreign countries. The sample size was attained through the Raosoft sample size calculator. The total estimated size is 3,854 with a 95% confidence level, ±1% margin of error, and 50% response distribution.

The participants were categorized by gender, location, age, and degree program. The participants included 1,823 students who were men, and 2031 students who were women. A total of 2,871 students were from the cities while 983 students were from villages or rural areas. The majority of the students were studying in undergraduate degree programs (2,853), followed by Masters (817), and PhD (184) programs.

As the students were studying in the online mode of teaching due to the restrictions of the pandemic and the implementation of social distancing, data were obtained by an online Campus Management Software (CMS) tool. The questionnaire was shared through the personal login IDs of the software. The software was secured for data confidentiality and the information was obtained as anonymous data. The participants were ensured that the information will only be used for research purposes. The language of the questionnaire was English. Students were informed about the objectives and purpose of the study by a description provided before the questionnaire. The consent of participants was taken and students who agreed were asked to fill out the questionnaire during the period of online enrollment. All the preventive measures were followed during the data collection.

### Data Collection Instrument

After a thorough literature review, a self-administered questionnaire was developed following the questionnaires of those studies. The draft of the questionnaire was approved by the senior scientists of the institute. After the review, the questionnaire was finalized. A pilot study was performed on 40 students of different degree programs to know the difficulties faced by participants. Participants of the pilot study were able to understand and attempt the questionnaire comfortably. A questionnaire was prepared which was comprised of three parts (i) knowledge about the transmission of COVID-19 disease, knowledge about the prevention of COVID-19 disease, (ii) Attitude about the disease, and (iii) practices about the prevention of disease.

The knowledge section (i) contained 14 questions (K1-K14), the attitude section (ii) contained 7 questions (A1-A7), while the final section on practices (iii) comprised 10 questions (P1-P10). The questions on Knowledge and Practices had three options: Yes, No and Not Sure; though questions of Attitude contained four options: Yes, No, Maybe, and Not Sure. In the questions relating to knowledge, the correct option was scored as one incorrect options were scored as zero. In the attitude segment, +1 point was given for true and −1 was given for the false answer. For questions on practices, two points were awarded for the yes option, one for sometimes and zero for no.

### Statistical Analysis

The collected data were analyzed with SPSS® software. The number of students answering the particular category variables was converted into a percentage frequency. We used the Chi-square test to analyze the answers of knowledge, attitude, and perception questions across different variables like gender, age, province, degree, number of family members, and area of residence. The statistical significance was considered at *p* < 0.05 while high significance was considered at *p* < 0.001.

## Results

**Figure 1:**
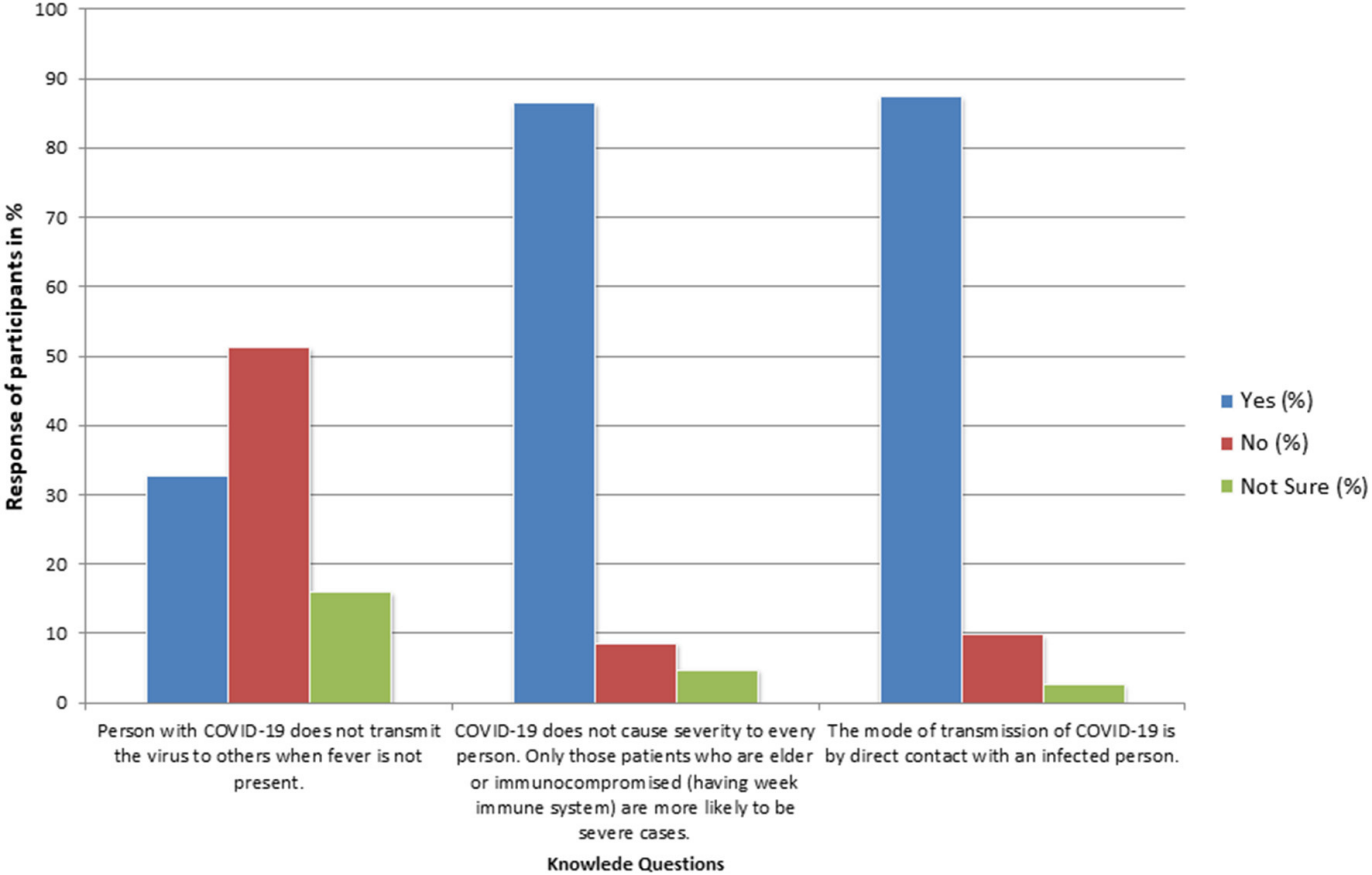
Percentage response of participants on important questions regarding the knowledge for COVID-19 (*N* = 3,854).

Total 97% of the students answered that the etiological agent of COVID-19 is a virus, and that it is a disease of the respiratory system (94%). The majority of the students had the wrong concept that direct contact (87%) can transmit the disease, although they also considered respiratory droplets (92%) to be the mode of transmission of COVID-19. A total of 87% of the students had the view that only older persons or those with weak immunity had a risk of severe symptoms. A total of 95% of students could identify the correct signs and symptoms of the COVID-19 (Supplementary Table 1). In total, 37% of students were not sure if COVID-19 can be transmitted by livestock, poultry, and their products, whole 33% of students ruled out the possibility of asymptomatic transmission (Figure 1).

**Figure 2:**
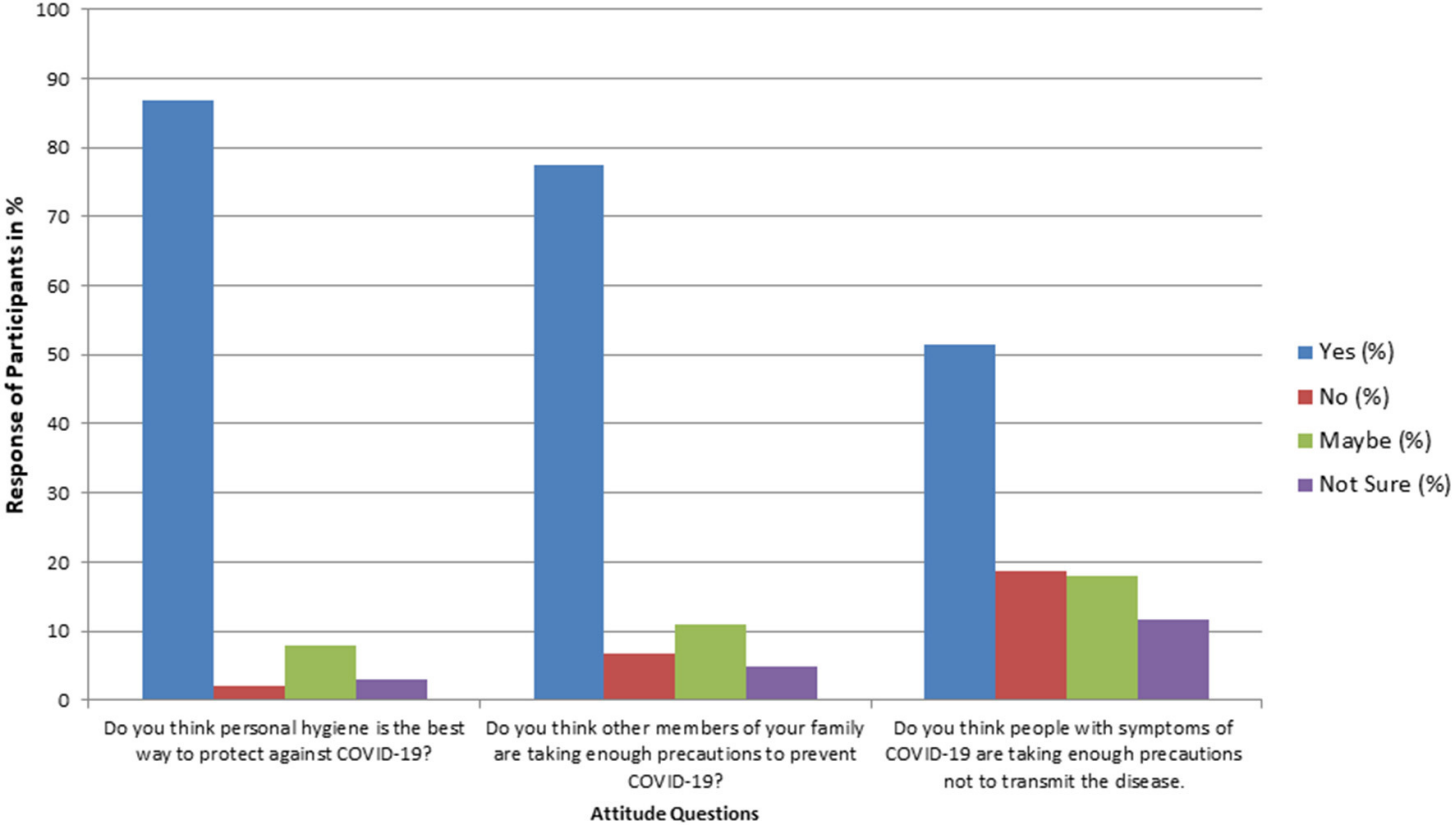
Percentage response of participants on important questions regarding the attitude for COVID-19 (*N* = 3,854).

**Figure 3:**
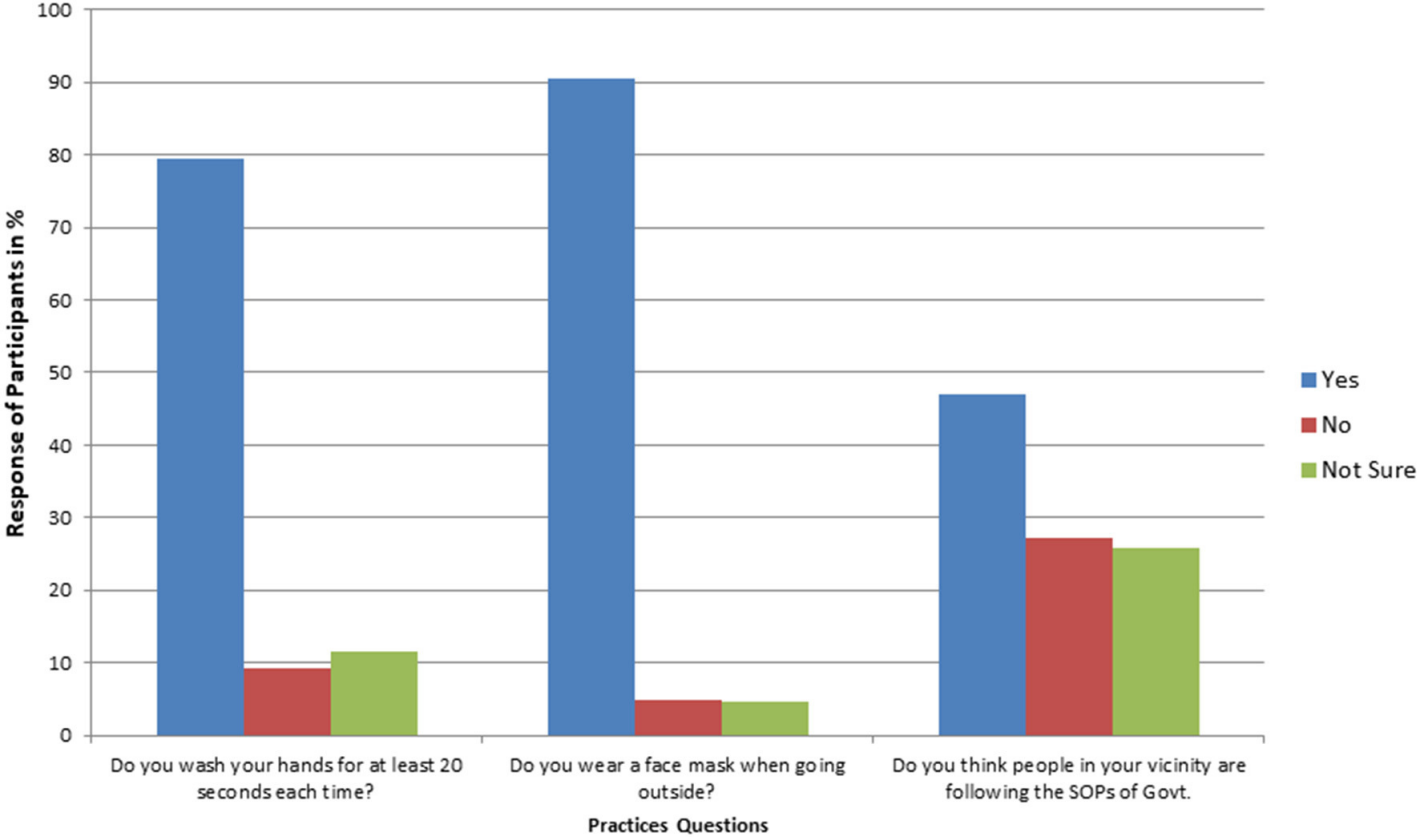
Percentage response of participants on important questions regarding the practices for COVID-19 (*N* = 3,854).

The majority of the students believed (97%) that personal hygiene is the best way to prevent the disease, however, they were not sure if they (>9%) and their family members (>15%) were observing the necessary hygienic practices (Figure 2). Nearly, 25% of the people in the locality of students were not taking enough precautionary measures. Many students kept visiting their relatives during the lockdown (45%) and their relatives kept visiting them at home (59%), and 10% of students did not wear a mask while visiting outside (Figure 3).

Among the Knowledge-based questions, the response of the students differed significantly for the categories of age and Provinces (*p* < 0.05). Students in the age groups of 25–34 and 35–44 had more adequate knowledge (90–91%) as compared to the age group of 18–24 (86%). Participants from KPK had less adequate knowledge (53%) than those of Punjab, Sindh, Balochistan, and Gilgit Baltistan (>80%). The Knowledge response of the participants did not differ significantly by the categories of gender, degree, family members, and area of residence (*p* > 0.05). However, there were fewer correct answers (85%) from the students who had more than 10 family members so the difference in knowledge was significant based on family members. There was not any significant difference in the knowledge of students from cities and villages (Supplementary Table 2).

For the attitude based questions, students knew that hygiene is essential for prevention but they had a varied response and were not confident that their family members and people in their locality were observing the precautionary measures. Male students showed significantly more precautionary measures (69%) than female students (66%) (*p* < 0.05). There was a more positive attitude toward the disease in the participants from ICT (77%), and the least positive response was received from the KPK (42%) (*p* < 0.05). No significant difference was found based on the students' degrees in regard to attitude response. Nevertheless, PhD students were following more precautionary measures as compared to Masters students, who were in turn observing more precautions than undergraduate students (*p* > 0.05) (Supplementary Table 2). To our surprise, students from villages mentioned that the community in villages was reporting cases more than in cities.

For the practices-based questions, people did not observe the practice of precautionary measures to the extent that they claimed in the knowledge and attitude-based questions. Nearly 92% of students were washing their hands frequently and regularly, however, only 79% were washing their hands for 20 s. Students wearing a face mask before going out totaled 90%. In all 55% were likely to visit relatives during the lockdown. Almost half of the community were not following the guidelines to prevent COVID-19 (Supplementary Table 1). The practices for participants from different categories did not differ significantly other than in the provinces. Practices were good in Baluchistan participants (70%) and poor from KPK (30%) (*p* < 0.001) (Supplementary Table 2).

## Discussion

The coronavirus disease 2019 (COVID-19) outbreak appeared in the fourth quarter of 2019 from Wuhan, Hubei province, China. Soon after its emergence COVID-19 had spread to most parts of the world and became a threat to public health. COVID-19 lead to significant socioeconomic damages to the whole world. More than 138 million cases have been reported with more than 2.9 million deaths globally till April 15, 2021. Along with the vaccine, strict precautionary measures are the only option to counter the rapid spread of the disease. COVID-19 vaccines are available now from international manufacturers in the developed world; however, they are less accessible in developing countries for mass vaccination for the reason that the government/administration is unable to cope with the cost associated with vaccine procurement from an international source. Therefore, strict precautionary measures need to be opted for in the communities of the developing world to stop the spread of COVID-19. In Pakistan, the first two confirmed COVID-19 cases were reported on February 26, 2020, in two individuals having a travel history to Iran (4). Till October 04, 2021, 1,252,656 cases and 27,947 deaths have been reported by Pakistan.

In this study, students were assessed for knowledge, attitude, and practices toward COVID-19 in UVAS, Lahore, Pakistan. A total of 3,854 students participated in this survey. Among these 1,823 were men and 2,031 were women. This survey showed that most of the students were well informed about COVID-19 and exhibited a proactive approach during the outbreak. This indicates an effective public health campaign of the local government to deliver public health knowledge in the community.

In our study results, 97% of the university students are aware that COVID-19 is a viral disease. However, a previous Pakistani study found that 59.3% of the survey participants from the general public know that SARS-CoV-2 causes COVID-19 (14). The difference in results of both studies could be due to the fact that the participants of the current study are students from medical, health, and allied sciences backgrounds. Therefore, their knowledge and understanding of COVID-19 is much better than that of the general population. However, our findings are justified by the studies from China and Japan that depict the critical role of education in the understanding of COVID (15, 16). Nearly 57% of the participants were satisfied with the government efforts to control the current pandemic. Interestingly, 50% of the participants believe that the government did not handle the pandemic crisis well, which is in line with one of the previous studies from the general population where only 48% of the participants were satisfied with the government efforts to control COVID-19 (14).

The majority of the participants in the current survey are well informed about the transmission of COVID-19. Similar knowledge about the transmission of COVID-19 was observed in KAP studies from Pakistan and China (14, 16). A majority of survey participants (95%) believe that frequent hand washing with soap and wearing masks are the best practices to avoid COVID-19 infection. The above findings are supported by three other Pakistani studies (17–19). We found 92% of the participants believe that only systematic and supportive treatment is considered best for patients who have recovered from COVID-19. Our results agree with the other study conducted in Nepal (20). The majority of the participants (73%) know that COVID-19 is not transmitted by animals, especially poultry, livestock, and their products. This result clarifies that the students do not believe in the rumors that animal products can be a source of COVID-19 viral spread. Most of the participants (95%) believe that social distancing and avoiding crowded palaces are a good way to avoid COVID-19 infection. Our results agree with previous studies conducted in China, the UK, South Korea, Indonesia, and the United Arab Emirates on KAP among students during various outbreaks (11, 15, 16, 21). In this study, social media was the leading source of information spread about coronavirus, outstripping print and electronic media. In the same line, social media remains the largest source of information in other studies from different countries (22, 23). This study has shown that participating women had more knowledge and were more aware of disease knowledge in comparison to men. Moreover, women proved superior to men in terms of practicing social distancing, hand hygiene, and wearing masks. A similar leading behavior in women was observed in the previous studies during various outbreaks (16).

Most of the participants were agreed that they were taking ample precautions to tackle this outbreak. But only half of the participants believed that other people in their vicinity were taking precautions, therefore nearly 50% of the participants believed that people in their vicinity are not taking precautions for COVID-19. This depicts that strict actions should be taken using law enforcement for the implementation of COVID-19 preventive measures. Moreover, only half of the participants agreed that COVID-19 patients were taking enough precautions during the course of the disease. Similar results were observed in a survey conducted on a different population (23). This shows that government needs to launch a campaign using social media about the precautions that should be taken by the COVID-19 patients. This will be helpful to reduce the spread of the disease in the community. Interestingly, 50% of the participants had visited the relatives during the lockdown period, which shows that people are not taking preventive measures seriously, which may lead to an increase in the number of cases in the near future.

Nearly 80% of the participants were confident that Pakistan can counter the disease spread, however, more than 30% of the participants were not satisfied with the approach of the government in handling COVID-19. Almost the same number of participants wanted the government to revise its strategies in controlling COVID-19 in the country. Currently, Pakistan has been adopting the strategy of smart lockdown, however the survey participants believe that a complete lockdown strategy is better than the smart lockdown strategy. Additionally, 90% of the participants believe that country will win the battle against COVID-19. In the culmination of the above survey findings, it is concluded that government should use social media platforms to spread knowledge about COVID-19 disease prevention in society and formulate a comprehensive program to deal with the COVID-19 pandemic.

The major limitation of this study is that the sample size is limited to the students of one government institution only, hence the results based on the current survey could not be generalized to the whole population of the country, however, current study findings can predict the thoughts of the university students. Moreover, the present study data will be helpful for the country to focus on the gaps that need to be strengthened to curb this disease.

## Data Availability Statement

The raw data supporting the conclusions of this article will be made available by the authors, without undue reservation.

## Ethics Statement

The studies involving human participants were reviewed and approved by Institutional Ethical Committee of UVAS, Lahore. The patients/participants provided their written informed consent to participate in this study.

## Author Contributions

TY and MN: conceptualization. MN and SR: designing of KAP Survey Form. ZA and MSa: acquisition of data. SR, MAs, and NM: analysis and interpretation of data. SR, MN, MAs, MSh, MAl, NM, RL, and TY: drafting the article and revision. All authors have approved the submitted version.

## Conflict of Interest

The authors declare that the research was conducted in the absence of any commercial or financial relationships that could be construed as a potential conflict of interest.

## Publisher's Note

All claims expressed in this article are solely those of the authors and do not necessarily represent those of their affiliated organizations, or those of the publisher, the editors and the reviewers. Any product that may be evaluated in this article, or claim that may be made by its manufacturer, is not guaranteed or endorsed by the publisher.

## References

[1] Tang JW Tambyah PA Hui DSC. Emergence of a novel coronavirus causing respiratory illness from Wuhan, China. J Infect. (2020) 80:350–71. 10.1016/j.jinf.2020.01.014 32001309 PMC7127306

[2] Gorbalenya A Baker S Baric R de Groot R Drosten C Gulyaeva A. Coronaviridae study group of the international committee on taxonomy of viruses. The species severe acute respiratory syndrome-related coronavirus: classifying 2019-nCoV and naming it SARS-CoV-2. Nat microbiol. (2020) 2020:3–4. 10.1038/s41564-020-0695-z 32123347 PMC7095448

[3] Guan WJ Ni ZY Hu Y Liang WH Ou CQ He JX. Clinical characteristics of coronavirus disease 2019 in China. N Engl J Med. (2020) 382:1708–20. 10.1056/NEJMoa2002032 32109013 PMC7092819

[4] Abid K Bari YA Younas M Tahir Javaid S Imran A. Progress of COVID-19 epidemic in Pakistan. Asia Pac J Public Health. (2020) 32:154–6. 10.1177/1010539520927259 32429679 PMC7240311

[5] Lauer SA Grantz KH Bi Q Jones FK Zheng Q Meredith HR. The incubation period of coronavirus disease 2019 (COVID-19) from publicly reported confirmed cases: estimation and application. Ann Intern Med. (2020) 172:577–82. 10.1101/2020.02.02.20020016 32150748 PMC7081172

[6] Tabary M Khanmohammadi S Araghi F Dadkhahfar S Tavangar SM. Pathologic features of COVID-19: a concise review. Pathol Res Pract. (2020) 216:153097. 10.1016/j.prp.2020.153097 32825963 PMC7334952

[7] Hakim AS Symae SM Shehata MM Sayed ATA. The battle with COVID-19: insight on external intervention and future vaccination. South Asian Journal of Research in Microbiology. (2020) 7:46–61. 10.9734/sajrm/2020/v7i230169

[8] Ali MY Bhatti R. COVID-19 (coronavirus) pandemic: information sources channels for the public health awareness. Asia Pac J Public Health. (2020) 32:168–9. 10.1177/1010539520927261 32429681 PMC7240310

[9] O'Connor C Murphy M. Going viral: doctors must tackle fake news in the covid-19 pandemic. BMJ. (2020) 369:m1587. 10.1136/bmj.m1587 32332066

[10] Puspitasari IM Yusuf L Sinuraya RK Abdulah R Koyama H. Knowledge, attitude, and practice during the COVID-19 pandemic: a review. J Multidiscip Healthc. (2020) 13:727–33. 10.2147/JMDH.S265527 32801735 PMC7407756

[11] Saefi M Fauzi A Kristiana E Adi WC Muchson M Setiawan ME. Survey data of COVID-19-related knowledge, attitude, and practices among Indonesian undergraduate students. Data Brief. (2020) 31:105855. 10.1016/j.dib.2020.105855 32607405 PMC7291994

[12] Al-Hanawi MK Angawi K Alshareef N Qattan AMN Helmy HZ Abudawood Y. Knowledge, attitude and practice toward COVID-19 among the public in the kingdom of Saudi Arabia: a cross-sectional study. Front Public Health. (2020) 8:217. 10.3389/fpubh.2020.00217 32574300 PMC7266869

[13] Habib MA Dayyab FM Iliyasu G Habib AG. Knowledge, attitude and practice survey of COVID-19 pandemic in Northern Nigeria. PLoS ONE. (2021) 16:e0245176. 10.1371/journal.pone.0245176 33444360 PMC7808653

[14] Rehman R Jawed S Ali R Noreen K Baig M Baig J. COVID-19 pandemic awareness, attitudes, and practices among the Pakistani general public. Front Public Health. (2021) 9:588537. 10.3389/fpubh.2021.588537 34178907 PMC8219954

[15] Hatabu A Mao X Zhou Y Kawashita N Wen Z Ueda M. Knowledge, attitudes, and practices toward COVID-19 among university students in Japan and associated factors: an online cross-sectional survey. PLoS ONE. (2020) 15:e0244350. 10.1371/journal.pone.0244350 33347488 PMC7751858

[16] Peng Y Pei C Zheng Y Wang J Zhang K Zheng Z. A cross-sectional survey of knowledge, attitude and practice associated with COVID-19 among undergraduate students in China. BMC Public Health. (2020) 20:1292. 10.1186/s12889-020-09392-z 32847554 PMC7447607

[17] Paul SS Deb SM Dey A Somvanshi SP Singh D Rathore R. 16S rDNA analysis of archaea indicates dominance of Methanobacterium and high abundance of Methanomassiliicoccaceae in rumen of Nili-Ravi buffalo. Anaerobe. (2015) 35:3–10. 10.1016/j.anaerobe.2015.06.002 26103451

[18] Salman M Mustafa ZU Asif N Zaidi HA Hussain K Shehzadi N. Knowledge, attitude and preventive practices related to COVID-19: a cross-sectional study in two Pakistani university populations. Drugs Ther Perspect. (2020) 36:1–7. 10.1007/s40267-020-00737-7 32395069 PMC7210795

[19] Faisal S Khotib J Zairina E. Knowledge, attitudes, and practices (KAP) towards COVID-19 among university students in Pakistan: a cross-sectional study. J Basic Clin Physiol Pharmacol. (2021) 32:681–6. 10.1515/jbcpp-2020-0436 34214368

[20] Sah GS Shrestha G Dhakal A Mulmi R Sapkota A Poudel S. Knowledge, attitudes, and practices of cancer patients towards COVID-19: a cross-sectional study in central Nepal. Cancer Manag Res. (2020) 12:10173–80. 10.2147/CMAR.S271910 33116872 PMC7573329

[21] Hasan H Raigangar V Osaili T Neinavaei NE Olaimat AN Aolymat I. Cross-sectional study on university students' knowledge, attitudes, and practices toward COVID-19 in the United Arab Emirates. Am J Trop Med Hyg. (2021) 104:75–84. 10.4269/ajtmh.20-0857 33236710 PMC7790059

[22] Dkhar SA Quansar R Saleem SM Khan SMS. Knowledge, attitude, and practices related to COVID-19 pandemic among social media users in J&K, India. Indian J Public Health. (2020) 64:S205–S10. 10.4103/ijph.IJPH_469_20 32496256

[23] Mahmood S Hussain T Mahmood F Ahmad M Majeed A Beg BM. Attitude, perception, and knowledge of COVID-19 among general public in Pakistan. Front Public Health. (2020) 8:602434. 10.3389/fpubh.2020.602434 33363092 PMC7755600

