# Supplementary material for: A Cross-Sectional Survey of Knowledge, Attitude, and Practices of University Students in Pakistan Regarding COVID-19
Source: Front Public Health. 2021 Nov 18;9:697686. doi: 10.3389/fpubh.2021.697686 (PMC8637527; doi:10.3389/fpubh.2021.697686)
Supplement: Supplementary file 1 [file Table_1.docx]

**Table1: Overall correct answer ratio of study participants**

| **Questions** | **Statement** | **Frequency** | **Percentage** | | |
| --- | --- | --- | --- | --- | --- |
| **Knowledge** | | **Correct Answer** | | | |
| K1 | COVID-19 is a viral infection. | 3742 | | 97 | |
| K2 | COVID-19 is also known as disease of respiratory system | 3632 | | 94 | |
| K3 | The mode of transmission of COVID-19 is by direct contact with an infected person | 3371 | | 87 | |
| K4 | COVID-19 also spread by the respiratory droplets of infected individuals | 3556 | | 92 | |
| K5 | COVID-19 does not cause severity to every person. Only those patients who are elder or immunocompromised (having week immune system) are more likely to be severe cases. | 3334 | | 87 | |
| K6 | Fever, cough, sneezing, runny nose, shortness of breath, fatigue and headache are the possible symptoms of COVID-19. | 3644 | | 95 | |
| K7 | Washing hands with soap and wearing masks is best practice to avoid COVID-19 infection. | 3661 | | 95 | |
| K8 | Only symptomatic and supportive treatment is considered best for the patients recovered with COVID-19. | 3551 | | 92 | |
| K9 | COVID19 is not transmitted by animals especially poultry, livestock and their products | 2799 | | 73 | |
| K10 | To avoid COVID-19 infection people must keep social distancing and also avoid going to crowded places | 3659 | | 95 | |
| K11 | Travel history of an infected COVID-19 patient is helpful in a clinical diagnosis | 3367 | | 87 | |
| K12 | Isolation and treatment of patients infected with COVID-19 is the best way to reduce the spread of COVID-19. | 3614 | | 94 | |
| K13 | The person who has contact with COVID-19 infected patient should be quickly quarantined in an appropriate place for 14 days. | 3609 | | 94 | |
| K14 | Person with COVID-19 does not transmit the virus to others when fever is not present. | 1260 | | 33 | |
| **Attitude** | | | | | |
| A1 | I think personal hygiene is the best way to protect against COVID-19? | 3351 | | 87 | |
| A2 | Do you think that staying at home is the best way to prevent the COVID-19 infection? | 3320 | | 86 | |
| A3 | I think I am taking enough precautions to prevent COVID-19? | 3137 | | 81 | |
| A4 | I think other members of my family are taking enough precautions to prevent COVID-19? | 2984 | | 77 | |
| A5 | I think other people in my locality are taking enough precautions to prevent COVID-19? | 1605 | | 42 | |
| A6 | I think infected people in my locality are reporting to the authorities? | 1795 | | 47 | |
| A7 | I think people with symptoms of COVID-19 are taking enough precautions not to transmit the disease? | 1983 | | 51 | |
| **Practice** | |  | | | |
| P1 | Do you wash your hands with soap frequently? | 3530 | | | 92 |
| P2 | Do you wash your hands for at least 20 seconds each time? | 3059 | | | 79 |
| P3 | Do you wear a face mask when going outside? | 3484 | | | 90 |
| P4 | Have you visited any relatives during lock down? | 1736 | | | 45 |
| P5 | Have any relative visited your home during lock down? | 2277 | | | 59 |
| P6 | Do you think that people in you locality have the symptoms of COVID but they are not reporting to authorities | 1244 | | | 32 |
| P7 | Do you think people in your vicinity are following the SOPs of Govt. | 1812 | | | 47 |
| P8 | Is there anyone in your family having the symptoms but not tested | 820 | | | 21 |
