# Supplementary material for: A Cross-Sectional Survey of Knowledge, Attitude, and Practices of University Students in Pakistan Regarding COVID-19
Source: Front Public Health. 2021 Nov 18;9:697686. doi: 10.3389/fpubh.2021.697686 (PMC8637527; doi:10.3389/fpubh.2021.697686)
Supplement: Supplementary file 2 [file Table_2.docx]

| Table 2: **Comparison of knowledge, attitude, practice, and misconception scores according to different variables.** |  | **Knowledge** | | **Attitude** | | **Practices** | |
| --- | --- | --- | --- | --- | --- | --- | --- |
|  |  | Adequate N (%) | Inadequate N (%) | Adequate N (%) | Inadequate N (%) | Adequate N (%) | Inadequate N (%) |
| Gender | Male (1823) | 1587 (87) | 236 (13) | 1262(69) | 561(31) | 1100(60) | 723(40) |
|  | Female (2031) | 1756 (86) | 275 (14) | 1334(66) | 697(34) | 1212(60) | 819(40) |
| P |  | 0.586932284 | | 0.019126941 | | 0.673933447 | |
| Age | 18 – 24 (3233) | 2788 (86) | 445 (14) | 2155(67) | 1078(33) | 1934(60) | 1299(40) |
|  | 25 – 34 (568) | 510 (90) | 58 (10) | 406(71) | 162(29) | 350(62) | 218(38) |
|  | 35 – 44 (44) | 40 (91) | 4 (09) | 31(70) | 13(30) | 25(57) | 19(43) |
|  | 45 or more (9) | 5 (56) | 4 (44) | 4(44) | 5(56) | 4(44) | 5(56) |
| p |  | 0.0035358180725 | | 0.05880729 | | 0.62431478 | |
| Province | Punjab (3741) | 3248 (87) | 493 (13) | 2519(67) | 1222(33) | 2247(60) | 1494(40) |
|  | Sindh (13) | 11 (85) | 2 (15) | 9(69) | 4(31) | 7(54) | 6(46) |
|  | Balochistan (10) | 9 (90) | 1 (10) | 7(70) | 3(30) | 7(70) | 3(30) |
|  | KPK (60) | 32 (53) | 28 (47) | 25(42) | 35(58) | 18(30) | 42(70) |
|  | Gilgist Baltistan (08) | 7 (88) | 1 (12) | 5(63) | 3(38) | 5(63) | 3(38) |
|  | ICT (22) | 19 (86) | 03 (14) | 17(77) | 5(23) | 13(59) | 9(41) |
| P |  | 0.0000000000749 | | 0.002096256 | | 0.00036447 | |
| Degree | Bachelor (2853) | 2459 (86) | 394 (14) | 1899(67) | 954(33) | 1710(60) | 1143(40) |
|  | Masters (817) | 721 (88) | 96 (12) | 564(69) | 253(31) | 492(60) | 325(40) |
|  | PhD (184) | 163 (89) | 21 (11) | 133(72) | 51(28) | 111(60) | 73(40) |
| p |  | 0.232783815 | | 0.14260724 | | 0.98559355 | |
| Family members | 1 - 3 (187) | 163 (87) | 24 (13) | 135(72) | 52(28) | 119(64) | 68(36) |
|  | 4 - 6 (2080) | 1804 (87) | 276 (13) | 1425(69) | 655(31) | 1254(60) | 826(40) |
|  | 7 -10 (1178) | 1031 (88) | 147 (12) | 787(67) | 391(33) | 707(60) | 471(40) |
|  | > 10 (409) | 346 (85) | 63 (15) | 250(61) | 159(39) | 233(57) | 176(43) |
| p |  | 0.5144536 | | 0.013870815 | | 0.445531553 | |
| City/village | City (2871) | 2496 (87) | 375 (13) | 1927 (67) | 944(33) | 1738(61) | 1133(39) |
|  | Village (983) | 847 (86) | 136 (14) | 670(68) | 313(32) | 575(58) | 408(42) |
| p |  | 0.53707322 | | 0.548593799 | | 0.259314053 | |
